# Characterization of a population of *Pelodera strongyloides* (Nematoda: Rhabditidae) associated with the beetle *Lucanus ibericus* (Coleoptera: Lucanidae) from Georgia

**DOI:** 10.21307/jofnem-2020-081

**Published:** 2020-08-31

**Authors:** O. Gorgadze, A. Troccoli, E. Fanelli, E. Tarasco, F. De Luca

**Affiliations:** 1 Institute of Zoology of Ilia State University, Tbilisi, 0162, Georgia; 2 Institute for Sustainable Plant Protection-CNR, Via Amendola 122/D, 70126, Bari, Italy; 3 Department of Soil, Plant and Food Sciences, Section of Entomology and Zoology, University of Bari “A. Moro”, via G. Amendola, 165/A, 70126, Bari, Italy

**Keywords:** D2 to D3 expansion domains, ITS, Maximum likelihood, Mitochondrial COI, Morphology, Phylogeny

## Abstract

During a field survey for entomopathogenic nematodes in Georgia, in the territory of Borjomi-Kharagauli, a nematode population was isolated from the body of single dead beetle of *Lucanus ibericus* Motschulsky 1845 (Coleoptera: Lucanidae). Based on morphological characters and molecular analyses, the nematode species is identical to *Pelodera strongyloides* (Schneider, 1860, 1866), being the first record of this species in Georgia. Morphometrics of the Georgian population agree with the original description, and females differ from males by very few characters. The Georgian population of *P. strongyloides* was molecularly characterized by sequencing the D2 to D3 expansion domains of the 28S rRNA gene and two new molecular markers as the ITS and the mitochondrial COI gene. Phylogenetic analyses revealed that all *P. strongyloides* sequences grouped together along with all other *Pelodera* species.

Nematodes of the genus *Pelodera* (Schneider, 1866) are widespread, mainly occurring as free-living saprophytes, which are usually found in the biological cycle in organic substances (Sudhaus and Schulte, 1988; Georgi and Georgi, 1991; Saari and Nikander, 2006). Some are facultative, opportunistic parasites, while others show a period of obligate parasitism in their life cycles. Several *Pelodera* species are also associated with termites (Carta et al., 2010) and rodents (Sudhaus et al., 1987; Casanova et al., 1996), but often are also reported as parasites of domestic animals and birds (Saari and Nikander, 2006). In Sudhaus’s (2011) revision of Rhabditidae, *Pelodera* is considered a genus comprising three major monophyletic groups the ‘*strongyloides*’, the ‘*coarctata*’and the‘*teres*’ groups. The ‘*strongyloides*’ group consists of six morphologically similar species associated with vertebrate and invertebrate hosts: the parasites *P. nidicolis* (Sudhaus and Schulte, 1986), *P. orbitalis* (Sudhaus and Schulte, 1986), *P. cutanea* (Sudhaus et al., 1987), *P. strongyloides* (Schneider, 1860; Sudhaus and Schulte, 1988), *P. termitis* (Carta et al., 2010), and *P. scrofulata* (Tahseen et al., 2014). Of particular interest is *P. strongyloides*, a small saprophytic nematode that usually reproduces on decaying organic matter, but there is a strain named *P. strongyloides dermatitica* (Talvik et al., 2005), the third-stage larvae of which are able to invade the skin of several animals causing dermatitis in dogs, sheeps, and humans (Sudhaus and Schulte, 1988; Georgi and Georgi, 1991; Tanaka et al., 2004; Saari and Nikander, 2006).

During a nematode survey in Georgia, a nematode species was recovered from the body of a dead individual of *Lucanus ibericus* (Coleoptera: Lucanidae) and identified morphologically as *P. strongyloides*. Considering the potential impact of *P. strongyloides* on human and animal health, accurate identification of this nematode species is necessary to provide proper management strategies. In the present study, a Georgian population of *P. strongyloides* recovered for the first time from *L. ibericus* and was fully characterized by morphology and morphometrics. Furthermore, the PCR amplification and sequencing of the D2 to D3 expansion domains of the 28S rRNA gene, the ITS, and the mitochondrial COI were carried out. The phylogenetic relationships of *P. strongyloides* from Georgia to other *Pelodera* species and Rhabditidae were also reconstructed.

## Materials and methods

### Nematode detection and isolation

*Pelodera strongyloides* was isolated in 2016 in the municipality of Borjomi, in the territory of the Borjomi-Kharagauli reserve, from the body of a single dead beetle belonging to *L. ibericus* (Coleoptera: Lucanidae). The beetle species was identified using morphological and morphometric criteria by entomologist of the Institute of Zoology of Ilia State University, Prof. Georgy Chaladze. Nematodes of different stages (L1 through adult), including females and males, were localized in the lipid tissue of the beetle organism. Insect cadaver with nematodes was placed on White trap (Kaya and Stock, 1997) and the emerging juveniles (J) were harvested and stored in 50 ml plastic tubes filled with wet polyurethane sponge at 4 to 6°C.

To obtain an isogenic population of this nematode species, one mature female and male were placed on water traps in the feeding area containing crushed *Galleria mellonella* L. (Lepidoptera: Pyralidae), *Tenebrio molitor* L. (Coleoptera: Tenebrionidae), and in agar peptonic plates. After 3 to 4 days, nematodes multiplied in the nutrient medium and nematodes at different stages, including females and males, migrated into water solution. As beetle-derived nutrition appeared to become scarce, the dauer larvae emerged and migrated in the water suspension. Extracted nematodes were morphologically and morphometrically studied in Tbilisi, in the laboratory of entomopathogens of the Institute of Zoology at Ilia State University (Georgia), whereas molecular analysis was carried out at the Institute for Sustainable Plant Protection in Bari, Italy.

### Nematode pathogenicity

Nematode pathogenicity of *P. strongyloides* was evaluated in comparison to *Steinernema carpocapsae* (Weiser, 1955) Wouts, Mráček, Gerdin, and Bedding, 1982, using last instar larvae of *G. mellonella*. Four Petri plates with 15 *G. mellonella* (last instar lavae) each were used for the tests. A distilled water suspension of 3 ml containing about 100 specimens/1 insect larva was inoculated into each Petri plate. Only distilled water was used as a positive control. Petri plates were incubated at 24°C in darkness and larval mortality checked at 3, 5, and 7 days after inoculation. Only 3.3% of the tested waxworm larvae were killed using *P. strongyloides* after 7 days of inoculation, whereas on day 5, 100% mortality of *G. mellonella* was observed with *S. carpocapsae*.

### Light microscopy and morphometrics

Measurements were conducted using a light biological research microscope (Motic®-DMB1). Nematodes were measured with an ocular micrometer and illustrated using a drawing (RA-4) tube.

**Figure 1:**
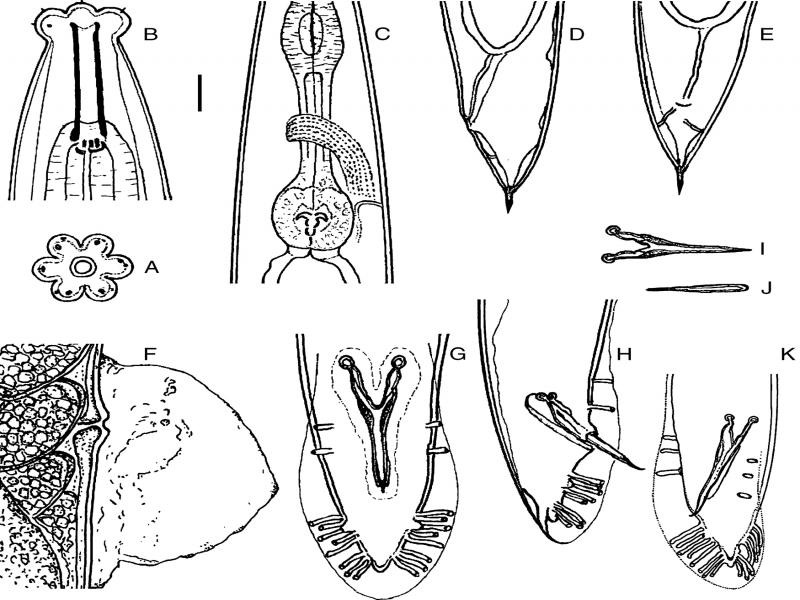
*Pelodera strongyloides* – Female. A: En face view; B: Anterior region (lateral); C: Pharyngeal bulb (lateral); D: Tail region; E: T region in ventral view; F: Vulval region in lateral view (sphincter, uterus, eggs). Male. G: Bursa (ventral); H: Tail (lateral); I: Aberrations of spicules, (ventral); J: Gubernaculum (ventral); K: Bursa (lateroventral). Scales (in μm): A, B = 10; C, D, E, F, I, J, H, K = 22.5.

**Figure 2:**
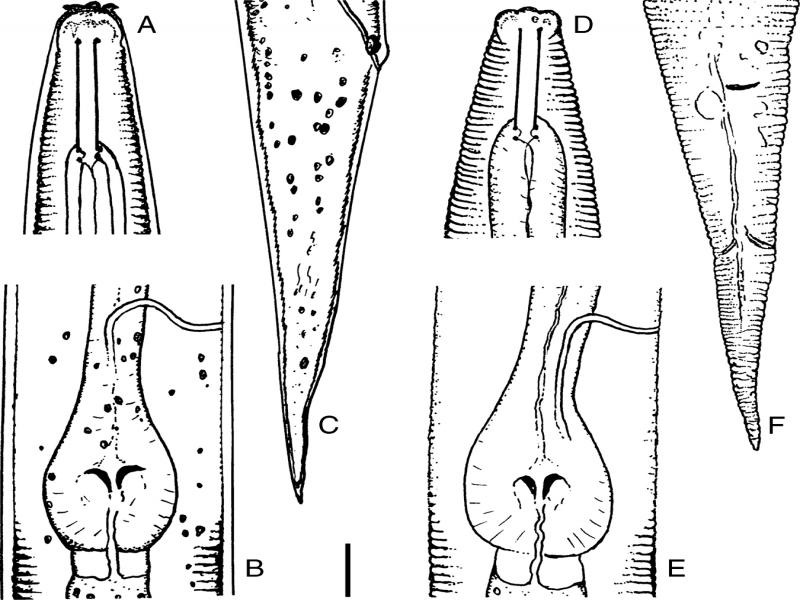
*Pelodera strongyloides* – Dauer larva. A: Anterior region, lateral; B: Pharyngeal basal bulb; C: Caudal region, in lateral view; third juvenile stage. D: Anterior region, in ventral view; E: Pharyngeal basal bulb; F: Caudal region, in ventral view. Scale bar = 10 μm.

**Figure 3:**
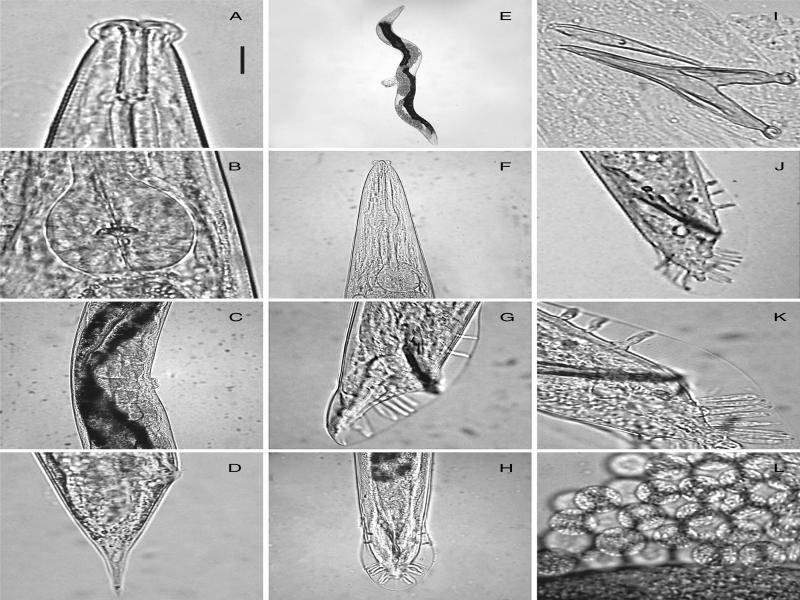
*Pelodera strongyloides* light photomicrographs – Female. A: Anterior end, lateral; B: Pharyngeal basal bulb; C: Vulval region, in lateral view; D: Tail region; E: Entire female; F: Pharyngeal region; Male. G: Tail, in lateral view; H: Tail, in ventral view; I: Excised spicules with gubernaculum; J, K: Tail, in lateroventral view; L: Cells of the spermatheca. Scales (in μm): A, B, I, K, L = 16; C, F, H = 50; E = 250; D, G, J = 22.5.

Drawings and photomicrographs of nematodes fixed on glass slides were taken using a digital video camera Genius (G-Shot) DV 1110 (Figs. 1-3). In total, 20 specimens from each stage (adults and 22 specimens of dauer juveniles) were randomly collected from *T. molitor* larvae. Nematodes were examined live or heat relaxed in Ringer’s solution at 60°C (Filipjev, 1934).

Nematodes were fixed in triethanolamine formalin (TAF) (Courtney et al., 1955) and processed to anhydrous glycerol for mounting (Seinhorst, 1959). Specimens were mounted on glass slides, and the coverslip was supported by glass rods to avoid flattening. Morphometric characters were selected according to Hominick et al. (1997) and reported in Table 1.

**Table 1.** Ratios and measurements (in µm) of heat relaxed specimens of *Pelodera strongyloides* collected from *Lucanus ibericus* in Georgia.

Character	Females	Males	3rd juvenile stage	*P. strongyloides* (Schneider, 1866)
*n*	20	20	22	11
Body length	1566 ± 120 (1353-1867)	1280 ± 100 (1080-1494)	663 ± 51 (566-772)	1629 (1142-2149)
Body width	96 ± 10 (75-112	62 ± 4.8 (55-70)	30 ± 2.4 (25-35)	107 (80-132)
Lip region diam	19 ± 1.6 (16-22	17 ± 1.5 (15-20)	–	–
Stoma length	31 ± 1 (30-32)	29 ± 2.2 (25-32)	24 ± 1.7 (22-30)	33 (30-38)
Stoma diam	5.1 ± 0.4 (5.0-6.2)	4.7 ± 0.4 (3.7-5.0)	2.6 ± 0.4 (2.5-3.7)	–
Anterior end to nerve ring	178 ± 6 (172-197)	155 ± 10 (137-175)	102 ± 6 (92-120)	–
Stoma length as % pharynx length	12.2 ± 0.4 (11-13)	13 ± 1.2 (11-15)	16 ± 0.8 (14-17)	–
Pharynx length	252 ± 5 (237-257)	219 ± 17 (192-250)	152 ± 6 (142-177)	–
Corpus length	144 ± 7 (135-162)	122 ± 10 (107-137)	87 ± 2 (80-92)	–
Corpus as % pharynx length	56 ± 2.3 (53-63)	55 ± 2 (50-59)	57 ± 2.5 (52-61)	–
Median bulb (MB) diam	27 ± 2.1 (22-30)	30 ± 4.9 (20-37)	11 ± 0.8 (10-12)	–
Terminal bulb (TB) diam	39 ± 4.1 (32-47)	34 ± 2.7 (32-40)	15 ± 0.6 (12-17)	36-50
MB diam. as % TB diam	77 ± 9.5 (54-88)	79 ± 6.8 (62-93)	–	–
Anterior end to excretory pore	236 ± 14 (212-270)	205 ± 13 (164-227)	130 ± 10 (110-150)	235 (183-295)
Ex. pore posit. as % phar. length	93 ± 5 (83-105)	93 ± 8 (75-109)	85 ± 6.7 (75-98)	–
Tail length	69 ± 11 (52-95)	53 ± 5 (45-62)	72 ± 4 (65-82)	–
Anal (cloacal) body width (ABW)	40 ± 3 (32-47)	37 ± 3 (32-42)	19 ± 1.8 (16-22)	–
Gonad length^a^	1285 ± 198 (995-1658)	956 ± 87 (747-1268)	–	890 (653-1157)
Gonad length as % body length	82 ± 15 (61-107)	72 ± 5 (66-82)	–	46-63
Gonad length. as % intestine leng.^b^	108 ± 23 (72-143)	93 ± 11 (69-110 )	–	–
Anterior gonad branch length	800 ± 104 (555-928)	–	–	–
Posterior branch length	546 ± 128 (290-794)	–	–	–
Testis flexure length	–	201 ± 49 (123-260)	–	–
Ant. flexure as % of ant. branch	33 ± 3 (26-42)	17 ± 4 (13-24)	–	–
Post. flex. as % of post. branch	27 ± 6 (13-34)	–	–	–
Sperm diameter	n.d.	5 ± 0.6 3. (7-7.5)	–	–
Egg length	60 ± 3.4 (55-67)	–	–	–
Egg diam	41 ± 4 (35-47)	–	–	–
Rectum length	38 ± 3.2 (30-42)	n.d.	24 ± 1.5 (22-25)	–
Rectum length/ABW	0.9 ± 0.1 (0.8-1.3)	n.d.	0.9 ± 0.1 (7-1.2)	–
Anus to phasmid distance	23 ± 1.8 (20-27)	n.d.	–	–
Anus to phasmid distance/ABW	0.6 ± 0.05 (0.4-0.7)	n.d.	–	–
Posit. phasmid as % tail length	35 ± 6 (27-48)	–	–	32-61
Spicule length	–	66 ± 3 (60-72)	–	55-82
Gubernaculum length	–	44 ± 2.2 (40-50)	–	–
Gubern. leng. as % spic. length	–	66 ± 4.5( 60-78)	–	–
*a*	16.3 ± 2 (13.5-20.3)	20.7 ± 1 (19-23.1)	22.3 ± 1.5 (19.3-25.8)	15.1 (12-18.7)
*b*	6.1 ± 0.4 (5.4-7)	5.8 ± 0.5 (5.1-7)	4.2 ± 0.2 (3.7-4.7)	6.2 (4.9-7.6)
*c*	23 ± 3.4 (17.6-32.2)	24.3 ± 2.8 (20.5-30.4)	9.1 ± 0.5 (8-10)	33 (26.3-37.9)
*c*′ (= tail length/ABW)	1.6 ± 0.6 (1.3-2.2)	1.3 ± 0.1 (1.2-1.7)	3.7 ± 0.3 (3.2-4.5)	–
V (vulva posit. in % body length)	57 ± 1.9 (51.8-59.1)	–	–	58 (55-64)

**Notes:** Measurements are in form: Mean ± standard deviation (range). ^a^From anterior to posterior flexure in the female; from cloaca to flexure in the male; ^b^distance from pharynx end to anus or cloaca, respectively.

### Cluster analysis

Diagnostics characters (Table 2) were used to compare *P. strongyloides* Georgian population with other species of the ‘*strongyloides* group’. Characters were ranked based on the principle of similarity. The state of the symbol ‘0’ was the most common sign, while the gradual increase in value represented a relative rarity and assumed an increase in deviation. The data matrix (Table 2) was prepared and analyzed using the Statistica-99 software, including cluster analysis.

**Table 2.** Data matrix for cluster analysis of the species of *Pelodera* ‘*strongyloides*’ group^a^.

Characters	1	2	3	4	5	6	7	8	9	10	11	12	13	14	15	16	17	18	19	20	21
*P. cutanea*	1	0	0	1	1	0	0	0	0	2	0	0	2	0	0	0	1	1	1	1	1
*P. nidicolis*	0	0	1	1	1	0	0	0	0	2	1	0	2	1	0	0	0	0	0	0	0
*P. orbitalis*	1	1	0	0	0	1	0	0	0	2	0	0	2	0	0	0	1	0	0	0	1
*P. strongyloides*	0	2	0	0	1	1	1	0	0	2	1	0	2	0	1	1	1	0	0	0	0
*P. strongyloides ex Lucanus ibericus*	0	2	0	0	1	1	1	0	0	1	1	0	0	0	0	1	1	0	0	0	0
*P. termitis*	0	0	0	0	1	1	?	0	1	1	0	1	1	0	1	1	2	0	0	0	0
*P. scrofulata*	1	1	1	1	1	0	0	1	1	0	0	1	0	0	0	0	1	0	1	1	1

**Note:** ^a^Data and explanation of state characters are in Tahseen et al. (2014).

### DNA extraction, PCR amplification, and sequencing

DNA was extracted from 15 individual specimens. Specimens were handpicked and singly placed on a glass slide in the lysis buffer (10 mM Tris-HCl, pH8.8, 50 mM KCl, 15 mM MgCl_2_, 0.1% Triton X100, 0.01% gelatine with 90 µg/ml proteinase K) and then cut into small pieces under a dissecting microscope, after which samples were incubated at 65°C for 1 hr, followed by deactivation of the proteinase K. PCR amplification, cloning, and sequencing protocols are described in detail by De Luca et al. (2004). The following primers were used for the ITS1-5.8S-ITS2 region using the forward primer TW81 (5′-GTTTCCGTAGGTGAACCTGC-3′) and the reverse primer AB28 (5′-ATATGCTTAAGTTCAGCGGGT-3′) (Joyce et al., 1994); for the D2 to D3 expansion segments of 28S rRNA using forward D2A (5′-ACAAGTACCGTGGGGAAAGTTG-3′) and reverse D3B (5′-TCGGAAGGAACCAGCTACTA-3′) (Nunn, 1992), the mitochondrial COI was amplified using COI-F1 (5′-CCTACTATGATTGGTGGTTTTGGTAATTG-3′) and COI-R2 (5′-GTAGCAGCAGTAAAATAAGCACG-3′) (Kanzaki and Futai, 2002). PCR amplifications were carried out in 100 µl volumes. PCR mix was added to each tube: 10 µl 10 x PCR buffer, 2 µl dNTP mixture (10 mM each), 2 µl of each primer 10 mM, 0.25 µl of Taq DNA polymerase (Roche), 73.5 µl of distilled water and 10 µl of crude DNA. Cycling conditions were 1 cycle of 94°C for 7 min followed by 35 cycles of 94°C for 50 sec, 55°C for 50 sec, and 72°C for 50 sec. The last step was 72°C for 10 min. PCR products were purified using the protocol listed by the manufacturers of Nucleospin Extract II (Macherey-Nagel, Duren) or QIAquick (Qiagen, USA) gel extraction kits and used for cloning or direct sequencing in both directions with the primers given above or M13 forward and M13 reverse primers. pGEM-T Vector System II kit (Promega) was used for cloning of PCR products. ITS-RFLP analyses were performed on PCR products from individual nematodes and digested with five units of the following restriction enzymes: *Alu* I, *Pst* I, *Dde* I, *Hinf* I, and *Rsa* I (Roche Diagnostics, Manheim, Germany). The restricted fragments were separated on a 2.5% agarose gel by electrophoresis. Gels were stained with gel red and were visualized on a UV transilluminator and photographed with a digital system.

The newly obtained sequences were aligned together with publicly available homologous sequences of *Pelodera* isolates using the computer program MAFFT (Katoh and Standley, 2013). Phylogenetic trees were reconstructed under the maximum likelihood (ML) criterion as implemented in MEGA v. 7 (Tamura et al., 2013). ML analysis was performed under a general time reversible and a gamma-shaped distribution (GTR + G) model for the 28S rRNA D2 to D3 domains, ITS, and mtCOI. Trees were bootstrapped 1,000 times to assess node support. Original sequences were deposited in GenBank under accession numbers: LR700238 to LR700241 for the D2 to D3 expansion domains of the 28S RNA gene; LR700237 for the ITS region; and LR700242 for the mitochondrial COI.

## Results

### Morphometrics of *pelodera Strongyloides* Georgian population

Measurements and figures are reported in Tables 1, 2 and Figures 1-3.

#### Description

*Female*: Body slightly is curved on ventral side, more rarely straight, after fixation, tapering at the extremities. Cuticle is faintly annulated and annuli 2.6 µm thick. Lateral lines are ornamented by weakly visible dots are observed throughout the body. Lip region is distinctly set-off, with six, prominent lips (Fig. 1A, B) and each lip with two small sensils in its upper and lower parts (Figs. 1B, 3A). Stoma is tubular, 5.5-6.2 times the width. Cheilostom is not cuticularized. Gymnostom is 62-74% the length of stoma. Metastegostom is isomorphic, slightly expanded with three, clearly pronounced teeth (Fig. 1B). Pharyngeal tissue (stegostom), which surrounds the main part of the bae of stoma, is 19 to 23% of stoma length. Pharynx is with slightly swollen corpus (ca. 56% of total pharyngeal length), long isthmus, and well developed, muscular, and nearly round (49-56 µm long, 32-47 µm wide) terminal bulb (Figs. 1C, 3B). Pharyngeal corpus is 1.1 to 1.4 times longer than the length of collar and basal bulb. Nerve ring surrounds isthmus at 66 to 78% of pharynx length. Excretory pore is located at 212 to 270 µm from anterior head with glandular cell difficult to observe by light microscopy (LM). Hemizonids can be observed in several copies just anterior to excretory pore. Intestine is granular, containing large polyhedral cells with pronounced nuclei. Rectum weakly cuticularized (Fig. 1D, E), 0.9 to 1.5 times long as the anal body diameter. Rectal cells are well-developed. Rectum is crescent shaped. The reproductive system is well developed, didelphic-amphidelphic. Oocytes are frequently arranged in several rows at the end of the distal dilated part of the ovary. Vagina is orthogonal to longitudinal body axis. Vulva is a transverse slit with sometimes bulging lips (Fig. 3C). Tail mostly is conoid to dome-shaped, tapering sharply in distal third, ending with a short spicate terminus with phasmids posterior to anus (Figs. 1D, E, 3D), with uniformly thick cuticle for 24 to 38% of tail length.

*Male*: Male is similar to female, but smaller in size and with narrower lips. Distance from the head to the excretory pore in males is 164 to 227 µm, in females 212 to 270 µm. Testis is monorchic with lateral reflection, about 1/5 of testis length up to flexure. Spermatocytes are ranging 3.7 to 7.5 µm in diameter. Spicules are transparent, light brown, elongated (Fig. 1I), joined and narrowing in distally ending in a slight hook (Figs. 1H, K, 3I). Spicules are adjoining for 58 to 75% of their length; capitulum is rounded (Figs. 1I, G, 3I). Gubernaculum is about 66% of spicule length, slender, widening proximally (Figs. 1, 3I). Bursa peloderan, with 10 pairs of genital papillae (Figs. 1G, H, 3G, H) (two pre-, and eight post-cloacal), arranged as: 1 + 1 + 1/5 + 3; GP1 and GP2 are sub-ventral (Figs. 1G, H, 3G, H). GP1 and GP2 are stout and shorter than other papillae; GP2 longer than GP1; GP3 and GP4 slightly dorsal; GP3 reaching the edge of bursa, and GP4 relatively short not reaching edge of bursa; GP5 sub-ventrally located on a small, swollen base and reaching the edge of bursa; GP6 smaller than previous three; GP7 sub-ventral; GP3-7 closely placed. GP8, GP9, GP10 closely placed with GP10 slightly smaller than the terminal trio. Distance between GP1 and GP2 is 9 to 12 µm; GP2 and GP3 29 to 33 µm; distances between the five GP (3-7) and the triplets (8, 9, 10) 3 to 5 µm. Bursal velum is faintly striated, ending in a crenate edge. Copulatory muscles are well developed, with 7 or 8 pairs of oblique bands. Tail is cone-shaped (Figs. 1G, 3H), shorter than female tail.

*Dauer larva: n* = 20; (Fig. 2A-C).

Body length = 618 ± 45 (530-676); pharynx length = 156 ± 8.0 (145-169); anterior end to excretory pore = 121 ± 7.5 (111-135); tail length = 75 ± 5.0 (72-78); *a* = 21.5 ± 2.0 (20.3-23.6); *b* = 3.8 ± 0.2 (3.5-4.5); *c* = 8.5 ± 2.1 (7.5-9.2).

Body is loosely sheathed by cuticle of second-stage juvenile, particularly visible by LM at mid-body and at anterior and posterior ends. The structure of the cuticle is barely visible under membrane and width of annuli 1.2 to 1.6 µm. Lateral fields are 5 to 6 µm wide at mid-body. Lip region is expanded, with incompletely merged lips, bearing sensillae (Fig. 2A). Metastomatal teeth are barely noticeable. Pharyngeal procorpus is 48 to 55% of total pharynx. Granular formations visible inside the body. Tails of second- and dauer stages long, and cone-shaped, tapering to pointed end (Figure 2C). Phasmids difficult to see.

#### Third juvenile stage (Fig. 2D-F)

Body is slightly longer and slender than that of dauer larva. Anterior body is end faintly annulated, width of five annuli at mid-body 9 to 12 µm; lateral fields are 7 to 8 µm wide at mid-body, extending from basal part of the stoma to the phasmids. Lips are merged (Fig. 2D); distal aperture is closed (Fig. 2F). Metastomatal teeth is not evident. Pharynx is longer than that of the dauer larva. Procorpus is 52 to 61% of pharyngeal length. Excretory pore is 75 to 98% of pharynx length. Intestinal cells are arranged to give a zebra-like appearance. Tail is conical (Fig. 2F), with finely rounded terminus. Phasmids are difficult to obseve by LM.

#### Variability

In male bursa, the standard number of papillae (10 papilla) and their location (1 + 1/5 + 3) display a range of variability similar to that reported for males of *P. cutanea* (Sudhaus et al., 1987). In several specimens of *P. strongyloides*, 11 pairs of papillae were observed (Figs. 1K, 3J, K), located three pairs before cloaca, and eight after cloaca; the arrangement of papillae is expressed by the following configuration: 1 + 1 + 1/5 + 3. Furthermore, in few females, the number of 26 eggs was observed in the uterus, larger than the (Fig. 3E), maximum of 19, characteristic of the species.

#### Type material

Collected from the cadaver of a beetle, *L. ibericus* (Coleoptera: Lucanidae), found on the soil surface in Borjomi-Kharagauli National Park, Georgia. Material collected at 798 m a.s.l., coordinates: 41°50′28.74″N, 43°23′06.51″E. Adult, dauer, and juvenile specimens (slides No. D-986-1007) deposited in Tbilisi, in the collection of the museum of the Institute of Zoology of the Ilia State University.

#### Molecular identification

The amplification of D2-D3 expansion domains of the 28S rDNA, the ITS containing region and the mitochondrial COI yielded single fragments of 556 bp, 611 bp, and 712 bp, respectively, based on sequencing.

**Figure 4:**
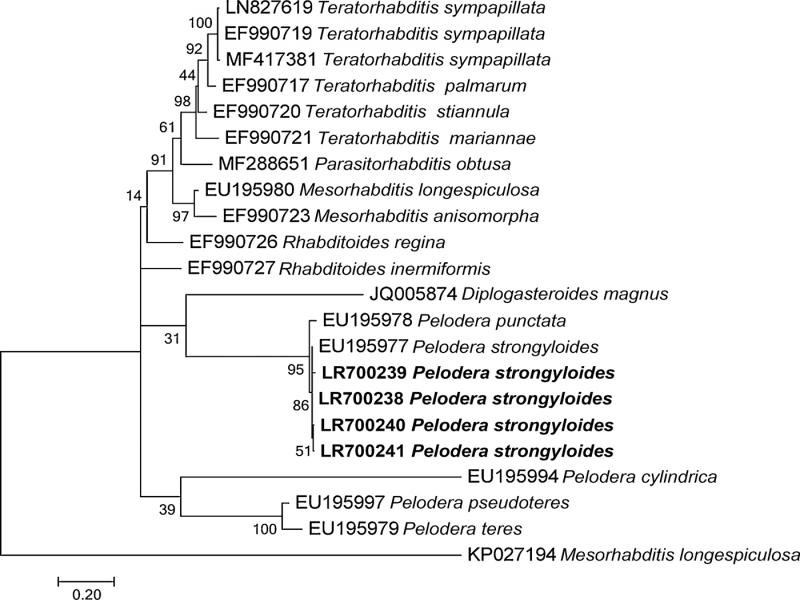
Phylogenetic tree of D2 to D3 expansion domains of 28S rRNA gene describing the evolutionary relationships among different geographical populations of Georgian *Pelodera strongyloides* under the maximum likelihood criterion. Values at nodes indicate bootstrap support.

Very low intra-individual and intra-population sequence variability in the D2 to D3 sequences have been observed (1-5 nucleotides). A BLAST search (Altschul et al., 1997) for D2 to D3 region showed a 100% identity with the homologous sequences of *P. strongyloides* (EU195977). The next-strongest match was *P. punctata* with 97% similarity. The topology of the inferred tree (Fig. 4) was congruent with those reported by other authors and all sequences of the Georgian population of *P. strongyloides* clustered into a well-supported group with that of previously sequenced *P. strongyloides* isolates. The *P. strongyloides* cluster also showed closely relationships with *P. punctata*, whereas the other *Pelodera* species clustered in different group.

The ITS sequence for Georgian isolate of *P. strongyloides* was determined for the first time in the present study and BLAST searches of the ITS sequence of *P. strongyloides* against the GenBank database revealed no corresponding sequences of *Pelodera* in the database.

**Figure 5:**
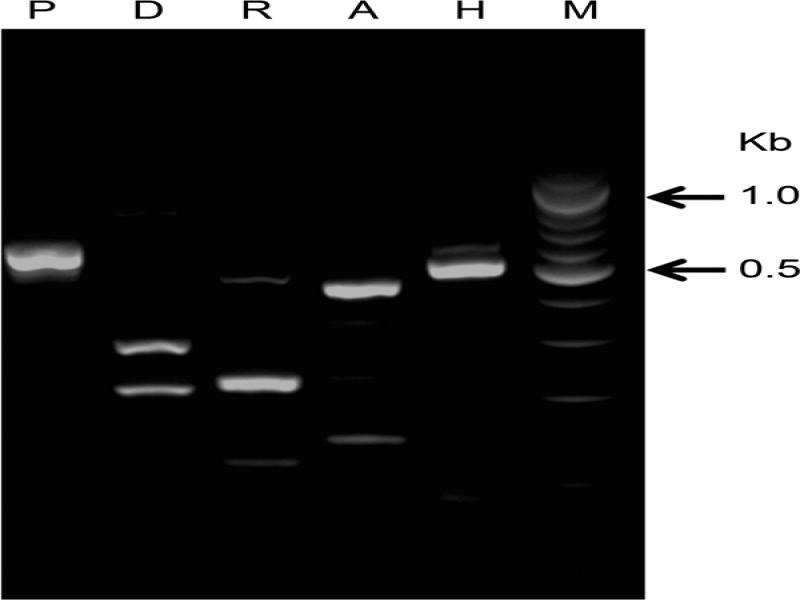
Restriction profiles of the ITS region of Georgian *Pelodera strongyloides* separated on 2.5% agarose gel. M: 100 bp DNA ladder; P) *Pst* I; D) *Dde* I; R) *Rsa* I; A) *Alu* I; H) *Hinf* I.

The ITS1 and ITS2 sizes were 186 bp and 217 bp, respectively, constituting the shortest ITS recorded for nematodes so far. PCR-ITS-RFLP patterns for *P. strongyloides* are given in Figure 4. Restriction of the PCR products by each of five enzymes: *Alu* I, *Dde* I, *Hinf* I, *Pst* I, and *Rsa* I, allowed us to determine the species-specific profiles for the Georgian *P. strongyloides* (Fig. 5).

The COI gene of one specimen sequenced here, as apparent from BLAST searches against the GenBank database, was also the first such sequence deposited in that database for a *Pelodera* isolate. The best-matching sequences found were *Micoletzkya palliati,* with 92% similarity (625/683 identity), *Teratorhabditis synpapillata,* with 91% similarity (645/711 identity; 1 gap), *Panagrellus redivivus,* with 90% similarity (638/710 identity).

## Discussion

In the present study, an approach integrating morphological and molecular sequence analyses was used to characterize a Georgian population of *P. strongyloides*. Until now, only two studies including sequences from *P. strongyloides dermatitica* (i.e. one of the 28S rRNA gene, one of the 18S rRNA gene, and two of the PolII gene) had been published to describe the phylogenetic relationships among rhabditids (Fitch et al., 1995; Kiontke et al., 2007). Our study reports the first record of *P. strongyloides* in Georgia and on a dead *L. ibericus* beetle, providing new examples of the extent to which *P. strongyloides* is associated with decaying materials, particularly beetle cadavers. Furthermore, we demonstrated that *P. strongyloides* is able to feed, grow and reproduce inside the bodies of the experimental insect hosts *T. molitor* and *G. mellonella*. Nevertheless, our pathogenicity assay revealed that *P. strongyloides* has only a low ability to infect and kill host larvae after 7 days of inoculation (only 3.3% mortality of waxworm larvae), while *S. carpocapsae* infected and killed 100% of waxworm larvae in 5 days. These observations suggest, in light of recent achievements (Blanco-Pérez et al., 2019; Zhang et al., 2019), that *P. strongyloides* can be considered a scavenger nematode, using insect cadavers as food.

The morphology and morphometrics (Tables 1, 2) of the Georgian population of *P. strongyloides* studied here were congruent with the original and subsequent descriptions of the species (Schneider, 1860; Sudhaus and Schulte, 1986; Sudhaus et al., 1987). By morphometric characters, major differences from the P. *strongyloides* type poulation consist of females having a shorter stoma length (30-32 vs 33-39 µm), a longer distance of excretory pore to anterior end (212-270 vs 79-108 µm), a different phasmid position (1/3 vs 1/2 of tail length), a shorter spike with respect to tail length (21-70 vs 18-20%, 1/4 vs 1/2 of tail length), and a sligthly smaller c value (17.6-32.2 vs 26.3-37.9), while males differ in having a GP5 on a small base, a trait apparently absent in the type population, and by a slightly shorter gubernaculum (40-50 vs 35-60 µm). These light morphological differences of Georgian *P. strongyloides* population with the original description of *P. strongyloides* and that of *P. strongyloides dermatitica* could be more conceivably interpreted in terms of host adaptation (in this case a cadaver of a lucanid beetle).

Molecular characterization of the new isolate also improved identification of this species with respect to other *Pelodera* isolates. First, restriction profiles obtained using six restriction enzymes, specifically of ITS region of *P. strongyloides,* are provided for the first time (Fig. 4) and can be used to easily differentiate among *Pelodera* species belonging to *strongyloides* group.

Second, the present phylogenetic analysis, using the D2-D3 expansion domains of the 28S rRNA gene, confirmed the grouping of the Georgian *P. strongyloides* population with *P. strongyloides dermatitica* with high support and the close relationships with *P. punctata*. Furthermore, our phylogenetic tree supports previous analyses that placed all *Pelodera* species along with *Teratorhabditis*, *Mesorhabditis*, *Parasitorhabditis* and *Rhabditoides* species (Kiontke and Fitch, 2005; van Megen et al., 2009). Together, our findings provide an improved comparative framework for *P. strongyloides dermatitica* and expand the known geographic and bionomic range of this peculiar species.
